# TouchView: Mid-Air Touch on Zoomable 2D View for Distant Freehand Selection on a Virtual Reality User Interface

**DOI:** 10.3390/s24227202

**Published:** 2024-11-11

**Authors:** Woojoo Kim, Shuping Xiong

**Affiliations:** 1 Division of Liberal Studies, Kangwon National University, Chuncheon 24341, Republic of Korea; woojoo.kim@kangwon.ac.kr; 2 Department of Industrial and Systems Engineering, Korea Advanced Institute of Science and Technology (KAIST), Daejeon 34141, Republic of Korea

**Keywords:** virtual reality, selection technique, mid-air interaction, human performance, user behavior

## Abstract

Selection is a fundamental interaction element in virtual reality (VR) and 3D user interfaces (UIs). Raycasting, one of the most common object selection techniques, is known to have difficulties in selecting small or distant objects. Meanwhile, recent advancements in computer vision technology have enabled seamless vision-based hand tracking in consumer VR headsets, enhancing accessibility to freehand mid-air interaction and highlighting the need for further research in this area. This study proposes a new technique called TouchView, which utilizes a virtual panel with a modern adaptation of the Through-the-Lens metaphor to improve freehand selection for VR UIs. TouchView enables faster and less demanding target selection by allowing direct touch interaction with the magnified object proxies reflected on the panel view. A repeated-measures ANOVA on the results of a follow-up experiment on multitarget selection with 23 participants showed that TouchView outperformed the current market-dominating freehand raycasting technique, Hybrid Ray, in terms of task performance, perceived workload, and preference. User behavior was also analyzed to understand the underlying reasons for these improvements. The proposed technique can be used in VR UI applications to enhance the selection of distant objects, especially for cases with frequent view shifts.

## 1. Introduction

Selection is one of the basic user interactions in virtual reality (VR) and 3D user interfaces (UIs) [1]. Poor selection techniques may severely degrade the quality of VR interactions, as selection is a prerequisite for the manipulation of virtual objects in general. Virtual pointing techniques (i.e., raycasting) are considered the most popular 3D selection techniques due to their simplicity and generality [2,3,4,5], resulting in better selection performance compared to other 3D selection techniques [1].

However, the selection of small or distant objects using raycasting has been known to be difficult. Raycasting is sensitive to natural hand tremors, as the selection occurs in mid-air, where there is no physical support for the hands [6], which leads to high error rates when selecting smaller targets [7,8,9,10,11]. A change in the tool’s orientation occurs through the action of the selection trigger (i.e., the Heisenberg effect on spatial interaction [12]), which also induces target misses, accounting for nearly 30% of all errors [13]. When the tracking noise becomes substantial, such as in vision-tracked freehand input, the negative influence of the positional and rotational jitters from the tracked input device on the selection accuracy can become non-negligible [14,15].

Many studies have proposed new ways to improve 3D selection. Some early research introduced volume-based pointing to enlarge the selection area itself [16,17], although it required disambiguation when multiple objects were inside the selection area. Disambiguation can be performed progressively by allowing users to specify the target [18,19,20,21] or heuristically by assigning scores to potential targets [11,22,23,24,25,26,27]. Another approach to improving 3D selection is to dynamically adjust the control–display ratio of the cursor depending on the precision requirement, which can be performed either manually [28,29] or automatically based on the user’s hand velocity [30,31,32]. Attempts to build and apply a model to correct the systematic mid-air pointing offset that is caused by humans’ limited inherent pointing accuracy [33] were also made [34,35].

Some techniques use an indirect approach whereby the user manipulates virtual objects by interacting with their copied representation, although this is typically used to enhance the manipulation rather than the selection of objects. World-in-Miniature [36] and Voodoo Dolls [37] were the first techniques to introduce the use of proxies for object manipulation. Indirect proxy techniques allow users not only to observe the world from different views, thus better understanding the layout of objects [38], but also to interact directly with the hands, thereby avoiding body distortion and disownership [39]. A recent advance in this approach includes interactions with proxies of space instead of objects [40], proxies of multiple virtual hands—Ninja Hands [41]—or even a handle connected by a tether to an out-of-reach object to decrease the distance to targets [42].

Adaptations have been made to benefit object selection with proxies by transforming 3D environments into 2D representations, as studies have shown that 2D selection can outperform 3D selection [43,44,45,46,47]. An early example of leveraging the benefits of 2D interaction in 3D space can be found in the Through-the-Lens metaphor [48], where the use of an additional viewport in the virtual world, in the form of a floating virtual panel, was proposed. Follow-up works have flattened targets and the surrounding environment onto various mediators, including a small virtual screen [49], a cylindrical virtual window displaying a 360° panoramic image [50], and a virtual viewport that reflects like a mirror [51]. The use of a tangible viewport utilizing a physical proxy has also been explored for selecting objects in VR [52] and handheld augmented reality [53,54,55,56]. Recently, the SmartVR Pointer technique further demonstrated the benefits of leveraging everyday devices like smartphones for VR interaction. By using smartphone tracking and gaze orientation, this technique enables users to perform VR selection and navigation tasks, providing a practical alternative to specialized VR controllers [57].

Meanwhile, recent advances in computer vision technology have introduced vision-based hand tracking using built-in cameras in consumer VR headsets, such as the Meta Quest series. This technology has now reached a level where it enables seamless interactions like object selection. This has greatly increased the accessibility of freehand mid-air interactions in VR without the need for gloves, markers, or other attachments. Not only do freehand mid-air inputs benefit from higher naturalness, intuitiveness, and unobtrusiveness, which are important values in VR, but they also reduce the cost and portability burden of ubiquitous computing by eliminating the need for additional wearable sensors or devices. In addition, when coupled with well-designed interaction techniques, there is great potential in terms of usability, as the hands are fine-dexterity tools that provide strength, precision, and expressiveness in our real-world actions [1].

Research on vision-based freehand interactions in VR is still scarce. Of the 48 papers included in a review article on the evaluation of VR object selection and manipulation, only eight (16.7%) investigated bare-hand interactions [58]. In VR ray-based pointing studies, VR controllers are used [59,60], or in the case of freehand interactions, an optical motion capture system is used to track markers attached to body parts, including the hands [34,35], to minimize the effect of tracking noise, thereby increasing internal validity in understanding human pointing behavior. Studies incorporating vision-based hand tracking noise have value for external validity, as this noise is unavoidable in real-world applications. This is particularly relevant given the current lack of comparative studies on Meta’s market-dominating Hybrid Ray technique [61]. Our approach, which includes realistic tracking noise, thus provides valuable insights into freehand interaction methods.

The study by Kim and Xiong [62] was the first to explore the full potential of the Through-the-Lens metaphor with the advantages of direct touch interaction and optimization of the interaction space in the freehand VR selection task. They proposed ViewfinderVR, a technique that allows users to configure the interaction space projected onto a virtual panel within reach and to select objects inside the panel through direct touch interaction. This technique highlighted the need for more intuitive and adaptable VR selection tools, as many existing methods lack the efficiency required for dynamic VR environments. They compared ViewfinderVR with Hybrid Ray, Meta’s implementation of freehand raycasting [61], using a 2D Fitts’ law-based test and found that ViewfinderVR induced significantly better performance and lower perceived workload. Another modern adaptation of the Through-the-Lens metaphor—although not concerning freehand interaction—is the work by Berwaldt et al. [63]. They introduced a method for enhancing VR interaction and navigation by employing multiple virtual cameras that project their views onto windows encircling the user, enabling users to simultaneously monitor, navigate, and engage with multiple occluded locations.

However, both of these approaches reveal a critical gap in VR selection techniques when frequent view adjustments are required. Earlier works require a considerable amount of additional time and effort to configure the panel to improve the object selection. This suggests that these techniques might not be suitable for scenarios where the view needs to be changed frequently, as the user has to reconfigure the panel whenever a target outside the panel view needs to be selected. To solve this problem, we propose the TouchView technique. A virtual panel with the Through-the-Lens metaphor is used, but the panel is always coupled with the user’s head movement and is large enough to cover the whole view, thereby significantly reducing the time needed for configuration. This study aims to fill the gap by offering a more flexible and responsive selection technique capable of supporting dynamic VR tasks. We conducted a user study to measure its performance against Hybrid Ray to understand the benefits and limitations of the proposed technique.

## 2. Techniques

### 2.1. Hybrid Ray

If any method of cursor movement that establishes the position of the cursor by a ray’s intersection with a surface or object in the distance is defined as generic raycasting, then there are the following variants: laser pointing, arrow pointing, image-plane pointing, and fixed-origin pointing [64]. Laser pointing specifies the ray directly according to the position and direction of a physical device. In contrast, arrow pointing works similarly to laser pointing but confines the use of the laser pointer to be aligned with the user’s eye. On the other hand, image-plane pointing allows the ray to be determined through the user’s eye location and another controllable point in space, while fixed-origin pointing relaxes image-plane pointing by directing one of the two points of the ray onto any fixed location rather than the eye.

In this study, Meta’s implementation of freehand raycasting—Hybrid Ray [61]—was used because it has been one of the most commonly used freehand ray-based pointing techniques and is the default selection method embedded in the current market-dominating VR headset, which occupied 72% of the market as of Q4 2023 [65]. Hybrid Ray is a variant of the fixed-origin pointing that uses a secondary position on the body to anchor the ray’s direction, thereby stabilizing the ray to minimize the negative influence of tracking jitter when using the vision-tracked freehand input. The optimal point of origin for this secondary position varies between shoulder and hip, depending on whether the user is standing or sitting [61], but Meta has not disclosed exactly how the secondary position is determined.

### 2.2. TouchView

**Figure 1:**
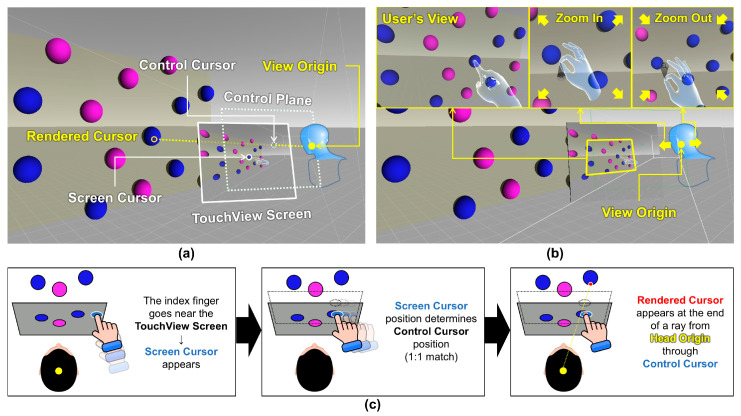
The working mechanism of TouchView: (**a**) main components, (**b**) zooming in and out the view, and (**c**) schematic explanation of the mechanism. Note: The components marked with dotted lines were invisible. A video demonstration of Hybrid Ray and TouchView performing the experimental task can be seen at the following link: https://vimeo.com/1019796139 (accessed on 31 October 2024).

Figure 1 illustrates the working mechanism of our proposed technique, TouchView. TouchView was designed as a technique that may be optimal for interacting with 2D UI elements with 3D planar panels, which may be a typical form of VR UIs. TouchView aims to aid the selection of distant objects by utilizing a virtual panel that shows the 3D environment behind it, taking advantage of the Through-the-Lens metaphor [48]. The virtual panel (TouchView Screen) located in front of the user’s head is large enough to cover the user’s whole view and follows the user’s head movement, making manipulation of the panel unnecessary. The view reflected on the panel can be magnified by moving the hand up or down while pinching with the index finger (Figure 1b), and the magnification of the view can be reset by turning the hand over and looking at the palm. When a pinch gesture occurs, a bubble-shaped indicator appears at the pinch location to signal entry into zooming mode. This indicator disappears when the index finger and thumb are separated. View magnification and object selection can be done by either hand.

TouchView consists of the following main components (Figure 1a):

- TouchView Screen: The virtual panel onto which targets are projected, allowing selection through indirect interaction. The TouchView Screen is constantly coupled with the user’s head movements and always covers the user’s entire field of view. The selection occurs when the index fingertip touches the surface of the TouchView Screen.
- Screen Cursor: The cursor that becomes visible when the user’s index finger approaches the TouchView Screen (Figure 1c). As the finger gets closer, the cursor shrinks and becomes more distinct. This cursor is positioned at the intersection between the TouchView Screen and an orthogonal vector from the user’s fingertip, indicating the point where selection can occur.
- Control Cursor: This invisible cursor is used to emit an invisible ray for target pointing. The Screen Cursor’s relative position on the TouchView Screen is mapped 1:1 to the Control Cursor, the movement of which occurs exclusively on the Control Plane (Figure 1c).
- Control Plane: The invisible interaction plane located in front of the view origin, where the Control Cursor moves. The Control Plane is constantly coupled with the user’s head movements, just like the TouchView Screen.
- View Origin: The origin point used to determine the view projected on the TouchView Screen. Initially, it is identical to the user’s head origin, but it moves forward when zooming in and backward when zooming out, based on the direction the user’s head is facing. The View Origin is constantly coupled with the user’s head movements.
- Rendered Cursor: The cursor for selection that appears as a red dot at the intersection point between the target object and the ray projected from the View Origin to the Control Cursor (Figure 1c). When selection occurs (i.e., when the user’s thrusting index fingertip passes through the TouchView Screen) while the Rendered Cursor is on the target surface, it results in a “target hit”. If the selection occurs while the Rendered Cursor is outside the target surface, it results in a “target miss”.

In this study, the panel was perpendicular to the front direction of the head and located 0.4m away and 0.13m below the head. This setting was determined based on various circumstances such as field of view, hand tracking range of the VR headset, and location to comfortably reach the panel [66,67,68]. The virtual hands were kept semi-transparent during the whole experiment to prevent the occlusion of the object behind the hands (Figure 1b).

## 3. Method

### 3.1. Participants

Twenty-three young Korean adults (16 males and 7 females) with a mean age of 24.3 years (SD = 3.3) and with normal or corrected-to-normal vision participated in the experiment. Twenty-two participants were right-handed, and one was left-handed. Twenty participants had prior experience with headset-based VR applications before this experiment, but 14 among them reported using the VR headset no more than once a year, indicating that the majority of participants were light VR users. Additionally, eight participants reported that they have experienced mid-air interaction with the tracked virtual hand in VR. All participants gave consent for the experiment protocol, which was approved by the University Institutional Review Board (IRB NO.: KH2021-009).

### 3.2. Experimental Settings

**Figure 2:**
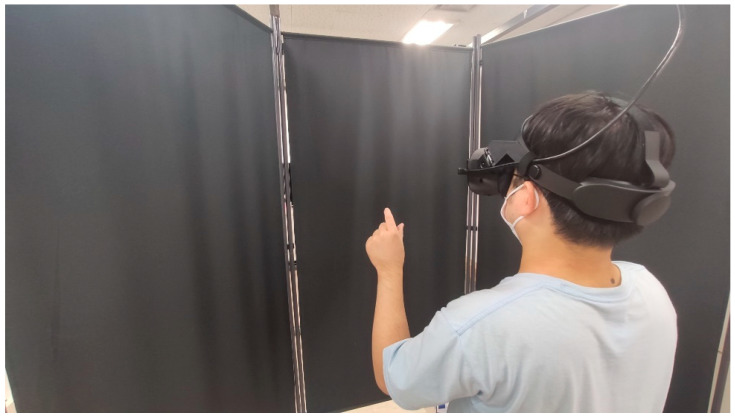
Experimental setup.

The participants were equipped with the Oculus Quest (resolution: 1440 × 1600 per eye; refresh rate: 72 Hz) VR headset. The headset was connected to a PC through Oculus Link via a compatible USB 3.0 cable. The PC was configured with an Intel Core i7-7700 processor running at 3.6 GHz, 16 GB of RAM, and an NVIDIA GeForce GTX 1080 Ti GPU, running Windows 10. Participants conducted the given task in a room while standing in front of a black screen fence (Figure 2). Hands were tracked in real-time by the four fisheye monochrome cameras embedded in the Oculus Quest headset [69], so the background was covered by a black screen fence to prevent any potential deterioration of tracking performance. The distance between the participant and the screen fence was 0.9m, and no physical interruptions were caused by the screen fence during the whole experiment. The virtual environment used for the experiment was developed using Unity 2019.4.15f1.

### 3.3. Experimental Design

**Figure 3:**
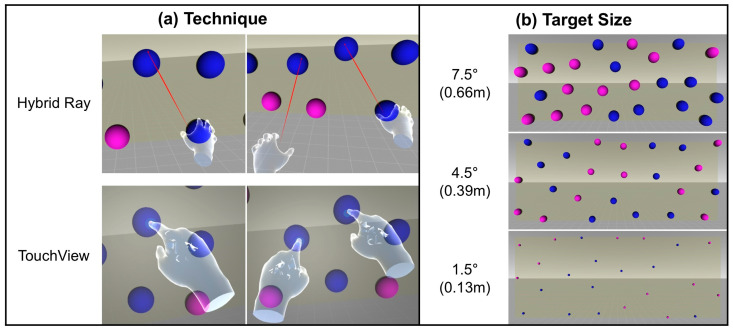
(**a**) Two selection techniques and (**b**) three target sizes of visual angle (diameter) used in this study. Note: A video demonstration of Hybrid Ray and TouchView performing the experimental task can be seen at the following link: https://vimeo.com/1019796139 (accessed on 31 October 2024).

The experiment followed a within-subject 2 × 3 full-factorial design with the following two factors (independent variables):

- Technique (2 levels): Hybrid Ray and TouchView (see Figure 3a)
- Target size (3 levels): 7.5°, 4.5°, and 1.5° (see Figure 3b)

The targets of 0.66 m, 0.39 m, and 0.13 m in diameter were used to represent large, middle, and small sizes, which were equal to the visual angles of 7.5°, 4.5°, and 1.5° from the distance of 5 m, respectively. It is worth noting that the distance from the user to the target ranged from 5 (center) to 8.12 m (corner), so the visual angles of actual targets were in the range of 4.62–7.50°, 2.77–4.50°, and 0.92–1.50°, accordingly. Target size and distance were determined based on the settings in previous VR target selection studies [7,47,70,71].

### 3.4. Experimental Procedure

**Figure 4:**
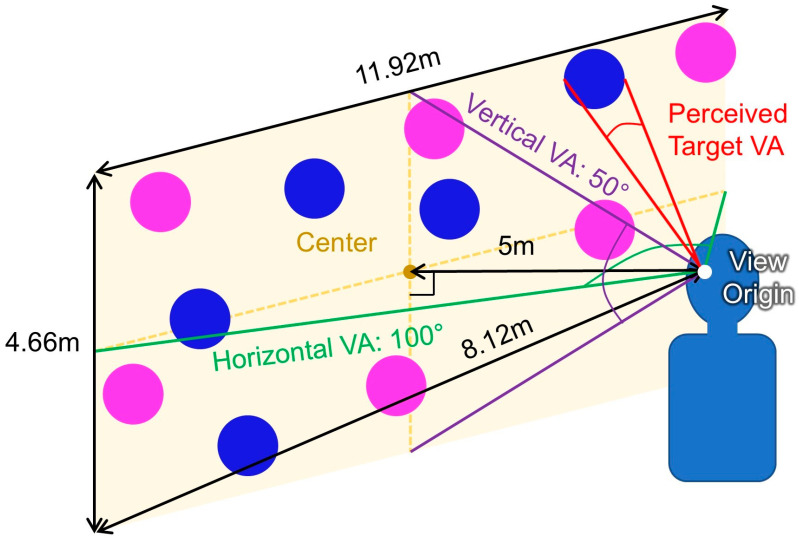
Target placement in the multitarget selection task. Note: VA indicates visual angle.

First, participants filled in a pre-test questionnaire asking about their demographic information and prior experience with VR. Then, they put on the VR headset and performed a multitarget selection task [72] for each of 6 experimental conditions in randomized order. In this task, 12 blue circular targets and 12 magenta circular distractors were placed randomly using a Poisson disk distribution on a rectangular plane 5 m away with a width × height of 11.92 m × 4.66 m, which corresponds to horizontal and vertical visual angles of 100° and 50°, respectively (Figure 4). A Poisson disk distribution was used to arrange the targets, ensuring an even yet random spread by maintaining a minimum distance between each point to prevent clustering. Here, targets were arranged on a planar space to simulate the selection on a UI. Participants were asked to select all targets as accurately and quickly as possible while prioritizing accuracy over speed. Upon selection, participants received auditory feedback: a click sound indicated a hit, or a beep sound signaled a miss. The selected target also changed its color to yellow. A sequence of trials consisted of 12 target selections, and five sequences were conducted for each of the experimental conditions. Each participant completed a total of 360 target selections, calculated as follows: 2 techniques × 3 target sizes × 5 sequences × 12 target selections.

Participants were asked to conduct the first sequence with their dominant hand only and the second sequence with both hands. Then, they could freely choose to conduct the remaining three sequences unimanually or bimanually based on their prior attempts. For TouchView, participants were allowed to change the magnification of the view at any time before or during the task. After all sequences in each test condition were finished, participants gave ratings to NASA-TLX [73], and then 30 s of rest, determined by a series of internal pilot tests, were given before moving on to the next test condition. Before the experiment ended, participants were asked to select the preferred technique per target size with reasons for their choice.

It should be noted that TouchView is primarily designed for use in VR UIs, where a typical scenario involves a large 3D planar panel with 2D UI elements floating in front of the user. Therefore, the experimental task in this study does not cover the varying depths of targets and cluttered environments that are often encountered in a general VR environment.

### 3.5. Data Analysis

Out of five sequences, the first two were considered practice and were thus excluded from the analysis. Data recording began when the participant selected the first target in each sequence. Therefore, a total of 198 target selections were analyzed per participant, calculated as follows: 2 techniques × 3 target sizes × 3 sequences × 11 target selections.

The following measurements were collected and analyzed (dependent variables):

- 2 performance measures: task completion time and miss rate (the number of misses divided by the total number of selections)
- 7 perceived workload measures (NASA-TLX ratings): mental demand, physical demand, temporal demand, performance, effort, frustration, and weighted rating
- 4 behavioral measures: dominant/nondominant hand movement, head movement, distribution of selections made by each hand, and target visual angle

Hand/head movements were defined as the length of the movement trajectory of the tracked dominant/nondominant hands and head. The target visual angle was defined as the visual angle of the target calculated based on the distance from the view origin to the target. All behavioral measures were recorded from the moment the first of the 12 targets was selected until the final target was selected.

The Shapiro–Wilk test for normality and inspection of Q-Q plots suggested that no measures deviated severely from the normal distribution. For all measures except the distribution of selections, repeated-measures analyses of variance (RM-ANOVAs) were conducted, with Bonferroni corrections applied for post hoc pairwise comparisons. The degree of freedom was corrected with the Greenhouse–Geisser correction if the p-value of Mauchly’s test was equal to or less than 0.05. Matlab R2019a, R 4.2.2, and “rstatix” R package were used to conduct all data processing and statistical analyses at a significance level of 0.05, and the effect size, in terms of generalized eta-squared ($\eta_{G}^{2})$ [74], was further calculated to assess practical significance. 

## 4. Results

We highlight a subset of significant results related to the technique for better clarity. The entire descriptive statistics and RM-ANOVA results are presented in Appendix A.

### 4.1. Performance

**Figure 5:**
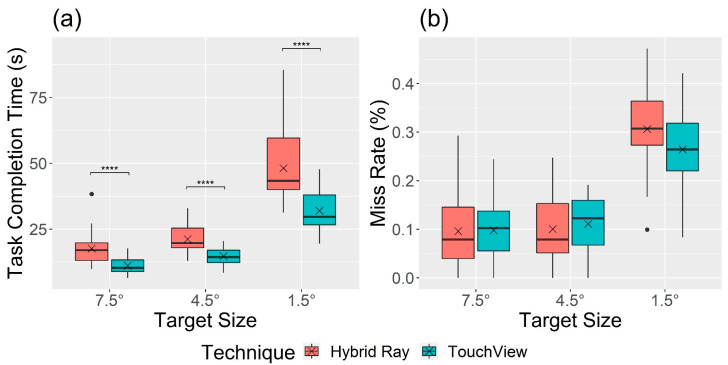
Boxplot of (**a**) task completion time and (**b**) miss rate by technique and target size. Note: The cross mark (×) indicates the mean, and the black circle mark (●) indicates values out of the interquartile range. The asterisk mark (****) indicates the significance of the post hoc analysis with a Bonferroni adjustment ( *p* < 0.0001). The same note applies to all other boxplots in the remaining text.

Figure 5 shows performance measures by technique and target size. A significant main effect of the technique was observed in task completion time ($F_{1,22}$ = 45.19, *p* < 0.001, $\eta_{G}^{2}$ = 0.290). The task completion time was shorter with TouchView (*M* = 19.3 s, *SD* = 10.5) than with Hybrid Ray (*M* = 29.0 s, *SD* = 16.8), but no significant difference in miss rate ($F_{1,22}$ = 0.66, *p* = 0.425, $\eta_{G}^{2}$ = 0.005) between Hybrid Ray (*M* = 16.8%, *SD* = 12.6) and TouchView (*M* = 15.8%, *SD* = 10.0) was found. A significant interaction effect of technique × target size was found for task completion time ($F_{1.2,26.51}$ = 9.88, *p* = 0.003, $\eta_{G}^{2}$ = 0.082) but not for miss rate ($F_{2,44}$ = 3.04, *p* = 0.058, $\eta_{G}^{2}$ = 0.025). According to the Bonferroni post hoc analyses, participants completed the task faster with TouchView in all target size conditions. Task completion time and miss rate with small targets increased compared to bigger targets in both techniques, but Hybrid Ray showed a larger increase compared to TouchView.

### 4.2. Perceived Workload

**Figure 6:**
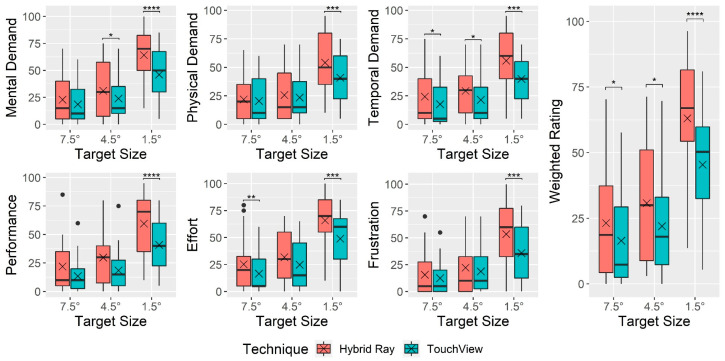
Boxplot of raw and weighted NASA-TLX ratings by technique and target size. Note: The cross mark (×) indicates the mean, and the black circle mark (●) indicates values out of the interquartile range. The asterisk mark (*) indicates the significance of the post hoc analysis with a Bonferroni adjustment (* *p* < 0.05, ** *p* < 0.01, *** *p* < 0.001, **** *p* < 0.0001).

Figure 6 indicates NASA-TLX workload ratings by technique and target size. A significant main effect of the technique was observed on all perceived workload measures, as follows: mental demand ($F_{1,22}$ = 24.24, *p* < 0.001, $\eta_{G}^{2}$ = 0.046), physical demand ($F_{1,22}$ = 6.45, *p =* 0.019, $\eta_{G}^{2}$ = 0.016), temporal demand ($F_{1,22}$ = 20.13, *p* < 0.001, $\eta_{G}^{2}$ = 0.049), performance ($F_{1,22}$ = 12.74, *p =* 0.002, $\eta_{G}^{2}$ = 0.082), effort ($F_{1,22}$ = 15.01, *p* < 0.001, $\eta_{G}^{2}$ = 0.054), frustration ($F_{1,22}$ = 10.78, *p =* 0.003, $\eta_{G}^{2}$ = 0.030), and weighted rating ($F_{1,22}$ = 21.74, *p* < 0.001, $\eta_{G}^{2}$ = 0.069). All NASA-TLX workload ratings indicated that TouchView was lower than Hybrid Ray, with the mean weighted rating of TouchView (*M* = 27.9, *SD* = 22.4) being 11.1 points lower than that of Hybrid Ray (*M* = 39.0, *SD* = 28.3). A significant interaction effect of technique × target size was found in mental demand ($F_{2,44}$ = 6.29, *p =* 0.004, $\eta_{G}^{2}$ = 0.017), physical demand ($F_{2,44}$ = 5.17, *p =* 0.010, $\eta_{G}^{2}$ = 0.015), frustration ($F_{2,44}$ = 6.66, *p =* 0.003, $\eta_{G}^{2}$ = 0.021), and weighted rating ($F_{2,44}$ = 4.47, *p =* 0.017, $\eta_{G}^{2}$ = 0.013). A similar tendency was found across all workload ratings, where the rating of Hybrid Ray was generally higher yet occasionally similar compared to TouchView at large and middle-sized targets, but the discrepancy became more obvious at small targets.

### 4.3. User Behavior and Preference

**Figure 7:**
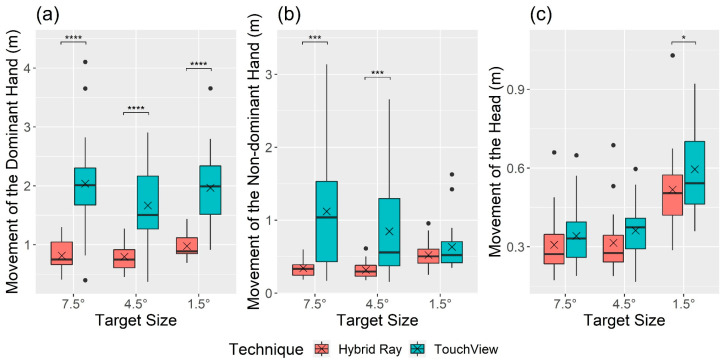
Boxplot of movement of (**a**) dominant hand, (**b**) nondominant hand, and (**c**) head by technique and target size. Note: The cross mark (×) indicates the mean, and the black circle mark (●) indicates values out of the interquartile range. The asterisk mark (*) indicates the significance of the post hoc analysis with a Bonferroni adjustment (* *p* < 0.05, *** *p* < 0.001, **** *p* < 0.0001).

**Figure 8:**
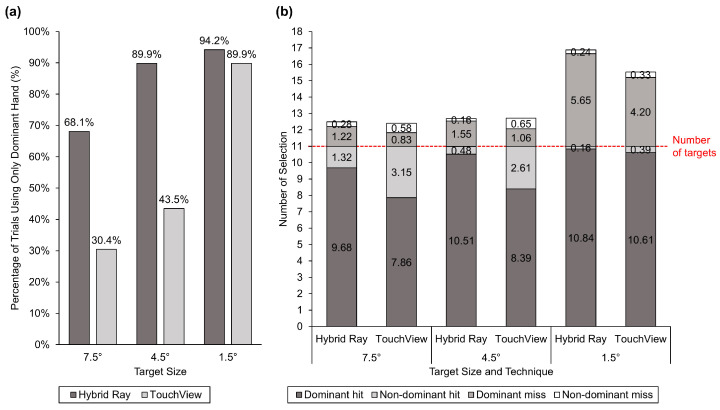
(**a**) Percentage of trials using only the dominant hand and (**b**) mean number of correct and incorrect selections (hit and miss) made by the dominant and nondominant hand.

There was a significant main effect of the technique on all user behavior measures (Figure 7 and Figure 8), as follows: movement of the dominant hand ($F_{1,\;22}$ = 71.68, *p* < 0.001, $\eta_{G}^{2}$ = 0.484), nondominant hand ($F_{1,\;22}$ = 23.12, *p* < 0.001, $\eta_{G}^{2}$ = 0.215), and head ($F_{1,\;22}$ = 7.60, *p =* 0.012, $\eta_{G}^{2}$ = 0.042), and target visual angle ($F_{1.01,22.14}$ = 10.54, *p* = 0.004, $\eta_{G}^{2}$ = 0.132). The hand and head movements of TouchView (dominant hand: *M* = 1.89 m, *SD* = 0.74; nondominant hand: *M* = 0.87 m, *SD* = 0.66; head: *M* = 0.43 m, *SD* = 0.17) were significantly longer than those of Hybrid Ray (dominant hand: *M* = 0.86 m, *SD* = 0.25; nondominant hand: *M* = 0.39 m, *SD* = 0.16; head: *M* = 0.38 m, *SD* = 0.16). For target visual angle, the adjusted target visual angle of TouchView (*M* = 4.22°, *SD* = 1.83) was larger compared to the original target visual angles of Hybrid Ray (*M* = 3.68°, *SD* = 2.01) and TouchView (*M* = 3.70°, *SD* = 2.03), where no significant difference was found between the latter two.

**Figure 9:**
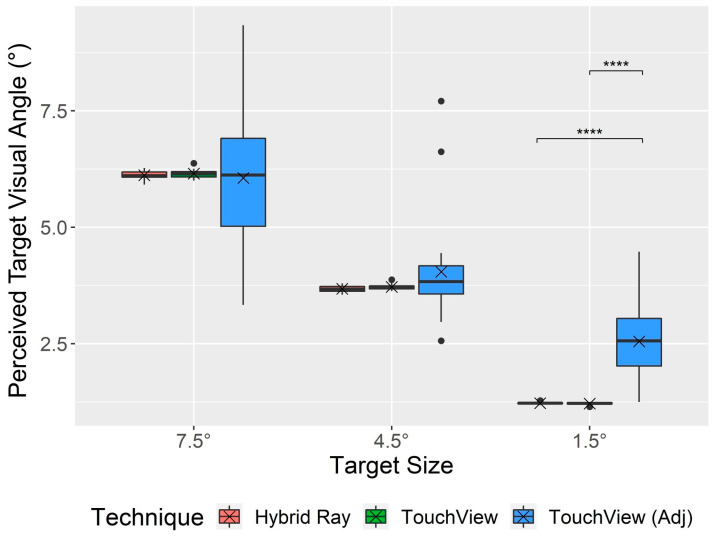
Boxplot of the target visual angle for Hybrid Ray and TouchView. Note: TouchView (Adj) indicates the target visual angle after adjustment by zooming in or out. The cross mark (×) indicates the mean, and the black circle mark (●) indicates values out of the interquartile range. The asterisk mark (****) indicates the significance of the post hoc analysis with a Bonferroni adjustment (*p* < 0.0001).

A significant interaction effect of technique × target size was found for the movement of the nondominant hand ($F_{2,44}$ = 11.43, *p* < 0.001, $\eta_{G}^{2}$ = 0.084) and target visual angle ($F_{1.86,41.03}$ = 11.40, *p* < 0.001, $\eta_{G}^{2}$ = 0.156). Movement of the nondominant hand with TouchView tended to decrease with the target size, showing no significant difference compared to Hybrid Ray at small targets (Figure 7). Figure 8 shows more detailed information about the usage of dominant and nondominant hands, which is in line with the hand movement. The percentage of trials using only the dominant hand almost doubled when Hybrid Ray was used compared to TouchView at large and middle targets but not at small targets. Likewise, selections with the nondominant hand were made more frequently with TouchView at large and middle targets, but the gap became less noticeable at small targets. The original target visual angle was consistent across both techniques, whereas the adjusted target visual angle was significantly larger than the original one at small targets (Figure 9). Notably, some participants intentionally reduced the target visual angle for selecting large and middle targets.

**Figure 10:**
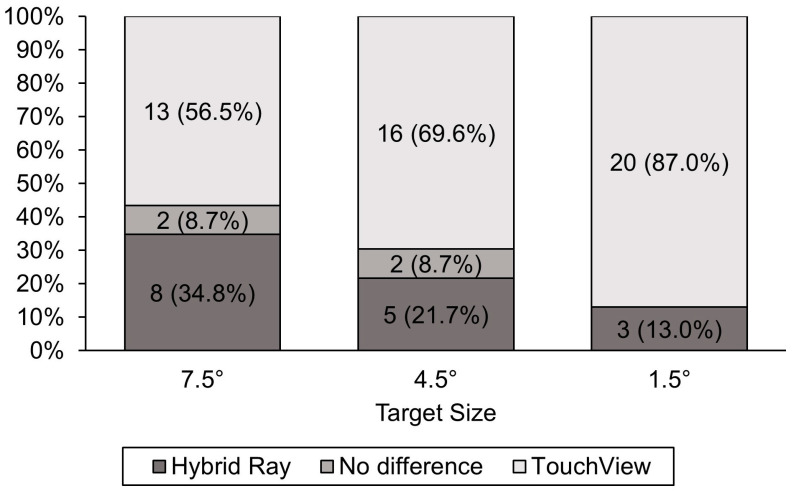
Frequency and percentage of the preferred technique by target size.

A larger number of participants preferred TouchView over Hybrid Ray in all target size conditions (Figure 10). However, preference differed depending on the target size, with five (21.7%), 11 (47.8%), and 17 (73.9%) more participants preferring TouchView over Hybrid Ray for large, middle, and small targets, respectively.

## 5. Discussion

### 5.1. Addressing Limitations of ViewfinderVR with TouchView

The major limitation of ViewfinderVR [62] was that it required a considerable amount of extra time and effort to configure the panel before object selection. This made ViewfinderVR impractical for cases where the view needed to be changed frequently since the user had to reconfigure the panel whenever the target beyond the panel view needed to be selected. TouchView simplified the configuration procedure by using a head-coupled panel large enough to cover the whole view, making manipulation of the panel unnecessary while maintaining the benefits by allowing magnification of the panel view. The average duration participants used to magnify the view while completing all three sequences was only 3.3 s (*SD* = 3.8), which was 84.4% shorter than the duration required for panel configuration with ViewfinderVR (*M* = 21.1s, *SD* = 11.8) [62].

### 5.2. Efficiency of Direct and Bimanual Touch Interaction

As the fundamental property of direct touch interaction transferred from ViewfinderVR to TouchView, similar results were expected to a certain extent. Direct touch interaction is known to require fewer mental resources compared to indirect methods, providing an intuitive way of interacting with objects [8,75,76,77]. Many participants (*P4, P5, P6, P9, P12, P13, P17, P21, P22*) who preferred TouchView over Hybrid Ray commented similarly, such as, “The way of interaction with TouchView was more direct and intuitive thus less demanding”. Direct input techniques have no intermediary to directly touch objects, unlike indirect input techniques that require additional cognitive processing of spatial translation between the body movement and the cursor movement [78]. Accordingly, the weighted workload rating of TouchView was 28.0–29.1% lower than Hybrid Ray. Although TouchView required users to move their upper body closer to targets to select them, thereby resulting in longer movements of hands and head (Figure 7), participants could hold up their arms for a shorter period when performing the same amount of tasks with TouchView. As physical demand is heavily influenced by the time that the upper arm muscles remain active for mid-air interaction [79,80,81], the rating of physical demand of TouchView turned out to be 6.0–24.5% lower than Hybrid Ray (Figure 6).

The directedness of interaction could further enable quicker task performance. Studies have found direct touch input allows users to perform the task faster and more accurately with higher satisfaction compared to indirect mouse input [72,82,83]. In this study, TouchView significantly outperformed Hybrid Ray in task completion time, lowering by 30.6–37.2% compared to Hybrid Ray, depending on the target condition. It is worth noting that the duration used for magnification of the view is included in the task completion time of TouchView since participants were allowed to magnify the view at any time during the task if needed. 

Bimanual interaction might also contribute to improving the selection speed of TouchView, as proven by research showing the speed advantages of bimanual touch over a pair of mice [72,84]. In this study, participants chose to select 16.6% more large targets and 19.4% more middle targets with the nondominant hand when using TouchView compared to when using Hybrid Ray, whereas they had difficulties in selecting small targets with their nondominant hand, and therefore chose to use the nondominant hand in only 4.3% more trials (Figure 8a). Accuracy demands of the task are known to influence both the performance and learning of unimanual and bimanual motor sequences [85,86]. In other words, the bimanual advantage is present for tasks that have relatively low accuracy requirements. As participants were asked to perform the task as accurately and quickly as possible, they likely chose to utilize bimanual advantage for large and middle targets that require low accuracy but not for small targets with high accuracy demands to avoid the bimanual disadvantage. This result aligns with previous studies that have reported a bimanual advantage and disadvantage associated with the task difficulty in selection tasks [85,86,87,88]. Nevertheless, the majority of targets were selected with the dominant hand (Figure 8b).

### 5.3. Drawbacks of View Magnification on Accuracy

Likewise, it was anticipated that TouchView would outperform Hybrid Ray in terms of miss rate, as several participants (*P8*, *P11*, *P15*, *P16*, *P23*) made comments such as, “An option to magnify the view was useful in improving accuracy for selecting smaller targets as the cursor jitter of Hybrid Ray was more sensitive to hand tremors and the occurrence of the pinch gesture for selection”. However, improvements in accuracy were minor in comparison with ViewfinderVR, as the miss rate of TouchView was not significantly lower than Hybrid Ray across all target sizes. This is because the view magnification of TouchView is inevitably accompanied by a decrease in the field of vision and an increase in the rate of change of the view, since the view was always coupled with the head movement. Consequently, excessive magnification could create more challenges in searching for targets out of the visual field and keeping the targets stationary within the view. Therefore, participants chose to adjust the target visual angle to be similar for large targets and larger by a small margin (8.6%) for middle targets to minimize such downsides but by a much bigger margin (109.0%) for small targets (Figure 9) where the benefits of magnification could surpass disbenefits. This indicates that TouchView is not a successor that can fully replace ViewfinderVR but rather a complement that can be used together with ViewfinderVR to cover the usage with frequent view shifts.

### 5.4. Limitations and Future Work

The proposed technique has some limitations. First, as TouchView projects the 3D environment onto a 2D screen, a slight distortion occurs in the view. Although distortion may not reach a level that significantly degrades selection performance, it may harm the naturalness of the interaction, as users can notice that the view shifts a bit differently from what they would expect when rotating their heads in the real world. Second, controlling the cursor of TouchView can be demanding for some users, as a few participants (*P3*, *P7*, *P20*) complained: “It required me to fully concentrate on keeping the rendered cursor inside the target while thrusting the finger”. This might be because the indication of the cursor location (i.e., the intersection point between the panel and the ray originating from the index fingertip and directed perpendicular to the panel) was less obvious to certain users. Future work could explore ways to further improve the proposed technique. 

In addition, it would be valuable to investigate whether the findings can be generalized to other options for the implementation of raycasting, other types of VR environments, and other VR headsets with different specifications. While the comparison with Meta’s Hybrid Ray can be very valuable in a practical sense, it is lacking in terms of understanding human pointing behavior and exploring better alternatives. Therefore, more diverse ray-based pointing methods such as finger, eye–finger, and head-based raycasting [35,89] can be further compared in future work. The experimental task used in this study was tailored for VR UIs, so it would be worthwhile to test the technique in a variety of VR environments in the future. For instance, TouchView omits depth information, which prevents the selection of occluded objects. This limitation is similar to that of Hybrid Ray, making both techniques less effective with occluded targets compared to those deliberately designed for such scenarios [90,91,92]. Finally, regarding the headset specifications, the accuracy of hand tracking might affect the result, although it should be noted that the VR headset used in this study—Oculus Quest—reported a comparable hand tracking accuracy in touch tasks to that of another commonly used hand tracking sensor, Leap Motion [93].

## 6. Conclusions

We have proposed a new technique, TouchView, to improve the selection of distant objects in VR UIs that can accommodate view shifts. TouchView allows users to configure the interaction space projected onto a virtual panel within reach and to select objects inside the panel through touch interaction. The virtual panel is large enough to cover the whole view and follows the user’s head movements, thereby removing the need for panel manipulation. A follow-up user study was conducted to evaluate and compare the proposed technique with Hybrid Ray using a multitarget selection test. Experimental results showed that for all investigated target sizes, TouchView resulted in shorter movement time and lower perceived workload compared to Hybrid Ray and has the advantages of the directness of interaction and target magnification inside the view. These benefits enable easier bimanual selection, contributing to shortening the task completion time. Our findings demonstrate that users can benefit from our proposed technique for distant selection in VR UIs due to direct mid-air touch interaction and customization of the interaction space.

## Data Availability

The data presented in this study are available upon request from the corresponding author.

## Appendix A

**Table A1:** *F*, *p*-values, and $\eta_{G}^{2}$ from RM-ANOVA results of the effects of technique and target size on performance, perceived workload, and user behavior measures.

**Measures**	Source of Variation								
	TQ			TS			TQ × TS		
	*F*	*p*	$\eta_{G}^{2}$	*F*	*p*	$\eta_{G}^{2}$	*F*	*p*	$\eta_{G}^{2}$
Task completion time (s)	45.19	*** <0.001	0.290	213.62	*** <0.001	0.689	9.88	** 0.003	0.082
Miss rate (%)	0.66	0.425	0.005	175.49	*** <0.001	0.597	3.04	0.058	0.025
Mental demand	24.24	*** <0.001	0.046	59.97	*** <0.001	0.305	6.29	** 0.004	0.017
Physical demand	6.45	* 0.019	0.016	36.80	*** <0.001	0.226	5.17	** 0.010	0.015
Temporal demand	20.13	*** <0.001	0.049	44.71	*** <0.001	0.213	2.81	0.071	0.008
Performance	12.74	** 0.002	0.082	53.48	*** <0.001	0.299	2.28	0.114	0.010
Effort	15.01	*** <0.001	0.054	60.50	*** <0.001	0.324	2.32	0.110	0.009
Frustration	0.78	** 0.003	0.030	47.68	*** <0.001	0.245	6.66	** 0.003	0.021
Weighted rating	21.74	*** <0.001	0.069	77.21	*** <0.001	0.350	4.47	* 0.017	0.013
D hand movement (m)	71.68	*** <0.001	0.484	3.80	* 0.030	0.037	2.03	0.143	0.019
ND hand movement (m)	23.18	*** <0.001	0.215	2.67	0.080	0.024	11.43	*** <0.001	0.084
Head movement (m)	7.60	* 0.012	0.042	80.76	*** <0.001	0.413	1.04	0.364	0.005
Target VA (°)	10.54	** 0.004	0.132	991.50	*** <0.001	0.888	11.40	*** <0.001	0.156

Note: TQ = Technique, TS = Target size, (N)D = (Non)dominant, VA = Visual angle; * *p* < 0.05, ** *p* < 0.01, *** *p* < 0.001.

**Table A2:** Mean (+ standard deviation) of performance, perceived workload, and user behavior measures by technique (All), and by technique and target size.

Measures	Hybrid Ray				TouchView			
	All	7.5°	4.5°	1.5°	All	7.5°	4.5°	1.5°
Task completion time (s)	29.0 (16.8)	17.7 (6.58)	21.2 (5.56)	48.2 (14.6)	19.3 (10.5)	11.1 (3.14)	14.7 (3.28)	32.0 (7.64)
Miss rate (%)	16.8 (12.6)	9.6 (8.0)	10.0 (7.1)	30.6 (8.4)	15.8 (10.0)	9.8 (6.2)	11.1 (5.8)	26.4 (7.8)
Mental demand	39.3 (30.1)	22.8 (23.2)	31.1 (25.2)	64.1 (24.9)	29.5 (24.3)	18.5 (19.7)	23.9 (20.8)	46.1 (23.5)
Physical demand	33.8 (27.0)	21.7 (20.9)	25.7 (21.7)	54.1 (26.3)	28.3 (22.9)	20.4 (20.7)	23.5 (20.6)	40.9 (22.5)
Temporal demand	36.5 (28.0)	24.3 (23.5)	29.3 (23.4)	55.9 (26.8)	26.3 (23.3)	17.6 (20.4)	21.5 (22.5)	39.8 (21.4)
Performance	37.1 (29.5)	22.0 (21.5)	29.8 (25.9)	59.6 (27.0)	24.3 (21.9)	13.5 (15.8)	18.5 (17.8)	40.9 (21.7)
Effort	40.9 (30.2)	25.2 (24.3)	31.7 (24.4)	65.9 (25.3)	30.1 (25.6)	16.5 (18.4)	24.8 (23.3)	48.9 (23.4)
Frustration	30.4 (30.2)	15.7 (21.1)	22.2 (24.4)	53.5 (30.5)	22.3 (23.5)	12.4 (16.8)	18.7 (21.3)	35.9 (25.7)
Weighted rating	39.0 (28.3)	23.2 (21.6)	30.7 (22.8)	63.1 (23.4)	27.9 (22.4)	16.4 (17.6)	22.0 (18.9)	45.4 (19.8)
D hand movement (m)	0.86 (0.25)	0.82 (0.27)	0.80 (0.24)	0.98 (0.22)	1.89 (0.74)	2.04 (0.83)	1.67 (0.72)	1.97 (0.62)
ND hand movement (m)	0.39 (0.16)	0.34 (0.13)	0.32 (0.11)	0.52 (0.16)	0.87 (0.66)	1.12 (0.85)	0.85 (0.63)	0.63 (0.32)
Head movement (m)	0.38 (0.16)	0.31 (0.12)	0.32 (0.12)	0.52 (0.15)	0.43 (0.17)	0.34 (0.11)	0.36 (0.10)	0.60 (0.17)
Original target VA (°)	3.68 (2.01)	6.12 (0.09)	3.68 (0.07)	1.23 (0.02)	3.70 (2.03)	6.15 (0.09)	3.72 (0.05)	1.22 (0.03)
Adjusted target VA (°)	NA	NA	NA	NA	4.22 (1.83)	6.06 (1.43)	4.04 (1.10)	2.55 (0.80)

Note: TQ = Technique, TS = Target size, (N)D = (Non)dominant, VA = Visual angle, NA = Not applicable
